# Stretching Reduces Tumor Growth in a Mouse Breast Cancer Model

**DOI:** 10.1038/s41598-018-26198-7

**Published:** 2018-05-18

**Authors:** L. Berrueta, J. Bergholz, D. Munoz, I. Muskaj, G. J. Badger, A. Shukla, H. J. Kim, J. J. Zhao, H. M. Langevin

**Affiliations:** 1 Osher Center for Integrative Medicine, Division of Preventive Medicine, Brigham and Women’s Hospital, Harvard Medical School, Boston, USA; 2 Dana Farber Cancer Institute, Institute, Department of Cancer Biology, Boston, USA; 3 Harvard Medical School Department of Biological Chemistry and Molecular Pharmacology, Boston, USA; 4 University of Vermont Department of Medical Biostatistics, Burlington, USA; 5 University of Vermont Cancer Center, Burlington, USA

## Abstract

There is growing interest in developing non-pharmacological treatments that could boost natural defenses against cancer and contribute to primary and secondary cancer prevention. Recent studies have shown that gentle daily stretching for 10 minutes can reduce local connective tissue inflammation and fibrosis. Because mechanical factors within the stroma can influence the tumor microenvironment, we hypothesized that stretching would reduce the growth of tumors implanted within locally stretched tissues and tested this hypothesis in a mouse orthotopic breast cancer model. Female FVB mice (N = 66) underwent bilateral injection of p53/PTEN double-null primary mouse mammary tumor cells into the third mammary fat pad. Mice were randomized to stretch vs. no stretch, and treated for 10 minutes once a day, for four weeks. Tumor volume at end-point was 52% smaller in the stretch group, compared to the no-stretch group (p < 0.001) in the absence of any other treatment. Cytotoxic immune responses were activated and levels of Specialized Pro-Resolving Mediators were elevated in the stretch group. These results suggest a link between immune exhaustion, inflammation resolution and tumor growth. Stretching is a gentle, non-pharmacological intervention that could become an important component of cancer treatment and prevention.

## Introduction

The last decades have seen a shift in approach to cancer biology and treatment. While cancer research originally focused on the neoplastic transformation of the cancer cells themselves, there is a growing interest in factors within the host that may influence cancer growth, such as angiogenesis, fibrosis, inflammation and immune dysregulation^1–5^. In addition to developing pharmacological treatments to influence these host factors, there has been interest in non-pharmacological treatments that could boost natural defenses against cancer and contribute to primary and secondary cancer prevention^6^. Among these, exercise has received a significant amount of attention due to the well-documented positive association between physical activity and survival in many cancer types^7,8^. However, to date, physical modalities are not used specifically to modify the neoplastic process. Animal studies of exercise in cancer models have yielded mixed results^9–17^. Furthermore, these studies have involved levels of vigorous aerobic exercise that can be difficult to achieve in cancer patients. On the other hand, gentle movement-based techniques such as yoga, tai chi and qi gong are popular and well tolerated among cancer patients for managing symptoms and improving mobility and well-being^18–20^. Stretching of tissues is a component of these techniques that has not been extensively studied, but could have important effects on the neoplastic process itself^6^. This is supported by a growing body of research pointing to the importance of mechanical factors in the pathophysiology of many diseases, including cancer^21,22^. Our recent studies show that gentle daily stretching for 10 minutes can have profound effects on reducing local connective tissue inflammation and fibrosis in several rodent models via direct mechanical effects on the stretched tissues^23–26^. A growing body of research suggests that mechanical factors within the stroma can influence the growth of tumors^3,27^. We therefore hypothesized that stretching would reduce the growth of tumors implanted within locally stretched tissues and tested this hypothesis in a mouse model of breast cancer.

## Results

**Figure 1:**
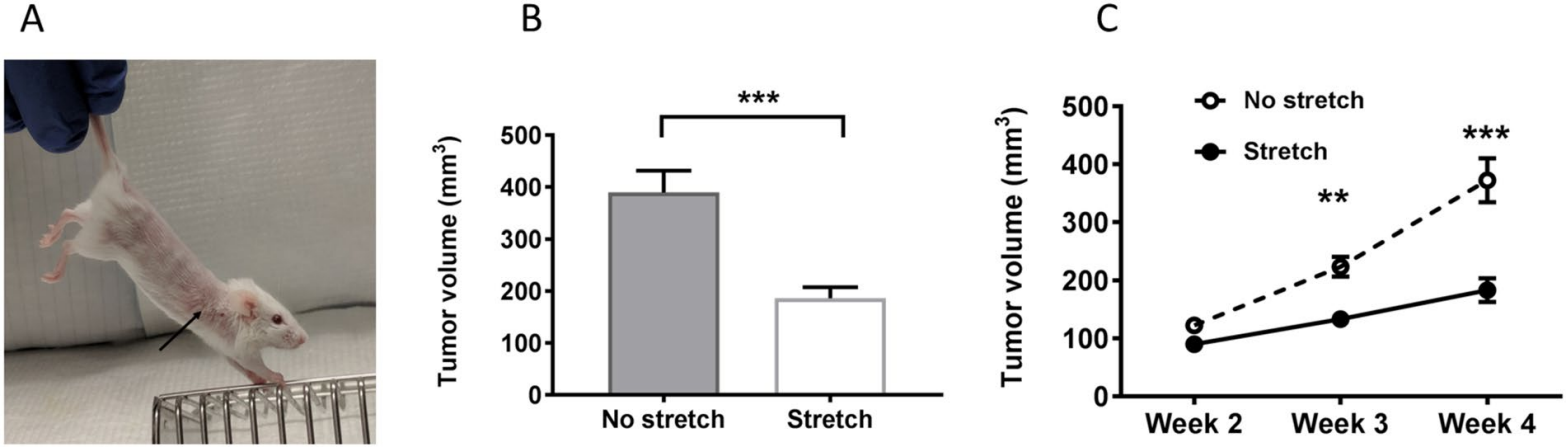
Stretching inhibits mammary tumor growth: (**A**) Stretching method. Arrow indicates location of tumor. (**B**) tumor volume (mean ± SE) at euthanasia (N = 33/group). (**C**) Tumor growth from weeks 2–4 (mean ± SE, N = 23/group). **p < 0.01, ***p < 0.001.

In order to investigate how stretching might affect breast cancer tumor growth, we adapted a well-established protocol for gentle stretching in mice^24,25^ in which mice are held by the tail and gently lifted, allowing the front paws to grasp a bar (Fig. 1A). We found in previous studies that, with minimal habituating, mice could hold this position comfortably for 10 minutes without struggling or vocalizing. In the current study, we injected immunocompetent FVB mice orthotopically with syngeneic p53/PTEN double-null (−/−) primary breast cancer cells into the third mammary fat pad located within the axillary region, and applied daily stretching for four weeks. Through this method, as illustrated in Fig. 1A, subcutaneous tissues of the front and back of the trunk are elongated due to simultaneous extension of the fore and hind limbs, which pulls on the subcutaneous and deeper tissues surrounding the tumor.

Tumor growth from weeks 2–4 was significantly slower in the stretched mice (repeated measured ANOVA group X time interaction p < 0.001, and tumor volume at euthanasia was 52% smaller in the stretch, compared with the no-stretch group (p < 0.001; Fig. 1B,C). Body weight at euthanasia was 3% lower in the stretch compared with the no-stretch group, however when tumor weight was subtracted from animal weight, the difference in animal weight between groups did not reach statistical significance (p = 0.05, Table 1). These results demonstrate for the first time that stretching reduces tumor growth in a mouse model of breast cancer.

**Table 1:** Study outcomes.

	N	No Stretch	Stretch	p value
Tumor volume (mm^3^)*	66	389 ± 243	186 ± 123	<0.001
Tumor weight (g)*	66	0.21 ± 0.17	0.14 ± 0.07	0.02
Animal weight (g)*^§^	66	21.3 ± 1.6	20.7 ± 1.0	0.05
Soluble cytokines (pg/ml)^#^				
IL-2	28	14.3 (7.5–26.6)	18.1(11.0–22.6)	0.81
IL-6	54	25.6 (4.5–91.4)	46.1 (21.1–95.5)	0.18
IL-10	54	102.1 (16.9–245.5)	159.3 (24.3–642.9)	0.35
TNF-α	54	15.5 (2.6–30.3)	25.1 (9.9–52.3)	0.18
INF-γ	54	29.0 (2.4–63.9)	39.9 (12.7–93.6)	0.24
SPMs (pg/ml)^#^				
RvD1	20	4.4 (1.5–21.7)	31.6 (10.9–61.2)	0.02
RvD2	20	10.9 (3.7–56.8)	95.4 (16.4–238.5)	0.04
Cellular Receptors				
Macrophages (%)*				
CD64^+^ (M1)	20	49.9 ± 24.8	51.0 ± 11.8	0.90
CD206^+^ (M2)	20	44.3 ± 22.1	45.2 ± 13.2	0.91
T lymphocytes (%)*				
CD3^+^	46	80.2 ± 17.9	77.1 ± 20.4	0.59
CD4^+^	46	77.0 ± 8.1	82.3 ± 4.7	0.10
CD8^+^	46	51.0 ± 30.0	43.4 ± 32.9	0.10
PD1^+^CD8^+^	46	20.7 ± 15.1	13.4 ± 7.38	0.04
Cytokines (%CD8^+^ cells)^#^				
IL-2	8	3.8 (2.3–6.7)	8.8 (7.3–9.1)	0.04
TNF-α	8	4.0 (2.1–6.2)	7.7 (5.5–7.9)	0.08
INF-γ	8	2.9 (0.9–5.8)	3.9 (1.2–6.4)	0.77

*Mean ± Standard Deviation.

^#^Median (Interquartile range).

^§^Total tumor weight was subtracted from the weight of each animal.

N represents total number of animals with equal number of stretch and no stretch.

CD64^+^ and CD206^+^ and CD3^+^ are expressed as a percentage of all leukocytes;

CD4^+^ and CD8^+^ are expressed as a percentage of TCR^+^ lymphocytes; PD1^+^CD8^+^ are expressed as a percentage of CD8^+^ lymphocytes.

**Figure 2:**
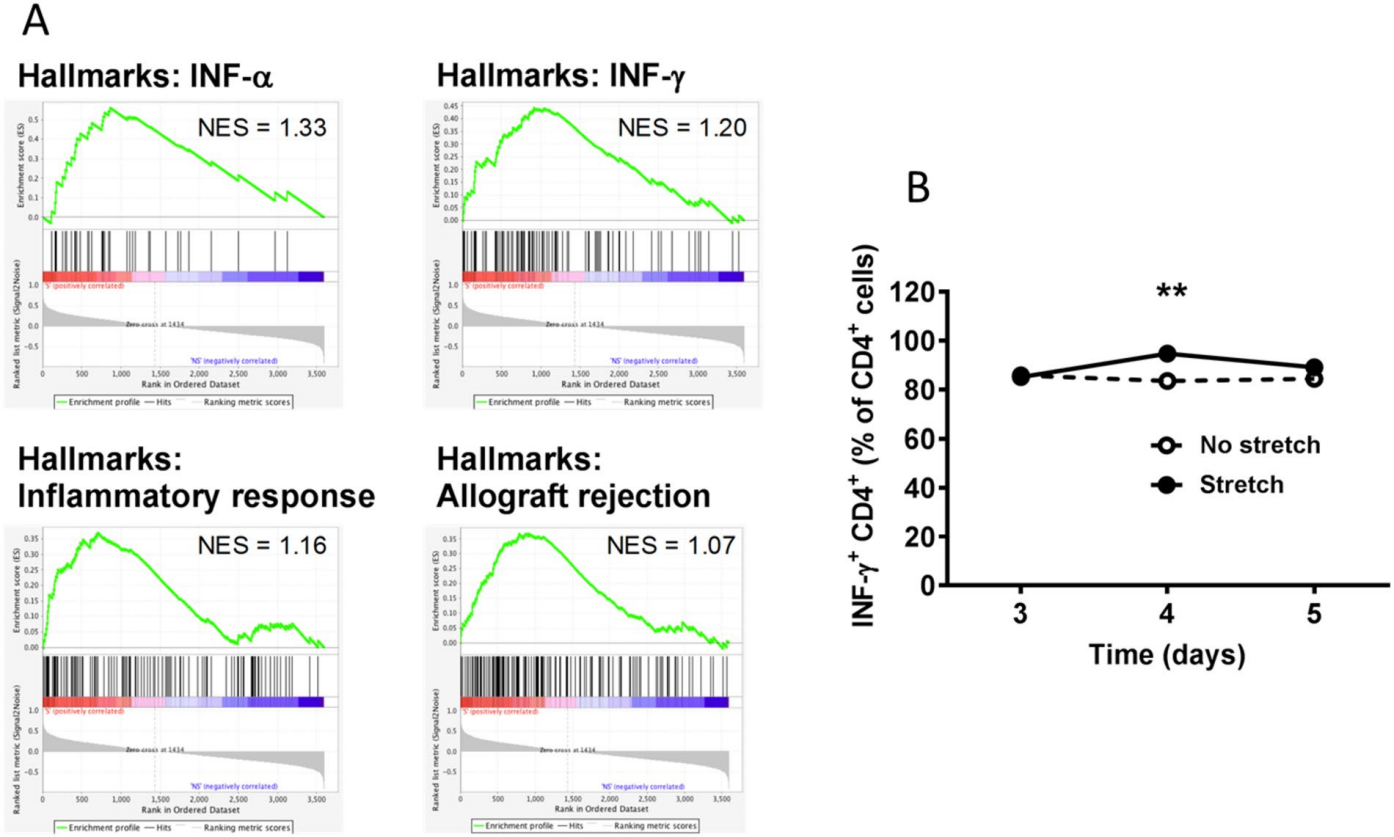
Stretching enhances immune response in cancer and chronic inflammation models: (**A**) Gene array analysis of whole excised tumors in p53PTEN−/− mammary cancer model, showing enrichment for immune-related gene expression signatures in stretch (red), compared with no-stretch (blue) (N = 5/group); NES: Normalized enrichment score. (**B**) Mouse subcutaneous carrageenan inflammation model, showing a stretch-induced increased in INF-γ^+^ CD4^+^ T-cells in whole inflammatory lesion during the transition between acute and chronic inflammation, 4 days after carrageenan injection (Mean ± SE, N = 3/group). **p < 0.01.

To investigate possible mechanisms underlying this reduction in tumor growth, we examined macrophage populations and soluble cytokines within whole tumors. We found no significant difference in the abundance of macrophages expressing CD64 (M1) and CD206/Arg (M2) between stretch and no-stretch groups (Table 1). We next examined tumor cytokine levels and found that, even though individual group differences were not statistically significant, overall levels of inflammatory mediators, including IL-2, IL-6, IL-10, TNF-α and INF-γ, were up-regulated in the stretch group (Table 1). To further investigate the effect of stretching on the immune response against tumor cells, we conducted gene array analysis of whole excised tumors. Gene set enrichment analysis (GSEA) revealed multiple gene signatures related to the immune response, including IFN-α, INF-γ, inflammatory response and allograft rejection, as the top enriched gene sets in the stretch group (Fig. 2A). To confirm this effect of stretching, we compared stretching vs. no stretching in a subcutaneous carrageenan-induced inflammation model, 3–5 days after injection, during the transition between acute and chronic inflammation. We found that stretching had a time-dependent effect (ANOVA p < 0.001) and increased the number of INF-γ^+^ CD4^+^ T-cells within the inflammatory lesion at the 4-day time point (p < 0.01, Fig. 2B). Because INF-γ is one of the key effector cytokines in cytotoxic immune responses, these results support a role for stretching in promoting TH-1 cytotoxic immunity.

**Figure 3:**
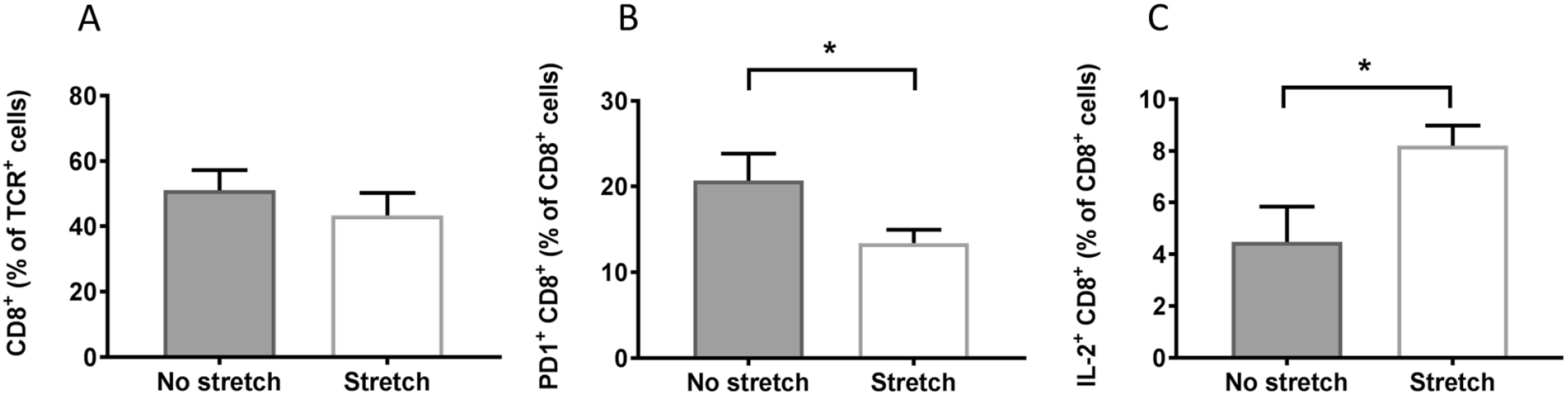
Stretching improves markers of T-cell exhaustion in 53/PTEN−/− mouse breast cancer model: (**A–C**) T-cell markers measured by flow cytometry. (**A**) CD8^+^ T-cells within tumor (Mean ± SE, N = 23/group). (**B**) PD-1^+^CD8^+^ T-cells within tumor (Mean ± SE, N = 10/group). C: IL-2^+^ CD8^+^ T-cells within axillary lymph node in response to stimulation with PMA/Ionomycin *in vitro* (Median ± SE, N = 4/group) *p < 0.05.

Importantly, impairment of cytotoxic immunity is a central mechanism underlying the phenomenon of T-cell exhaustion in cancer, and is characterized by a reduction in effector cytokines and the presence of inhibitory receptors on the surface of T-cells that promote tolerance to cancerous cells. Because stretching increased INF-γ, we hypothesized that stretching restores cytotoxic immunity and investigated T-lymphocyte populations involved in adaptive immunity against cancer. We first examined total numbers of CD3^+^, CD4^+^ and CD8^+^ tumor-infiltrating T cells, and found no significant differences between stretch and no-stretch groups (Table 1). Since cell number is not necessarily correlated with cell function, we next measured the sub-population of CD8^+^ T cells bearing the Programmed Death Receptor-1 (PD-1), one of the major markers of T-cell exhaustion. PD-1 expression in CD8^+^ T cells was lower in stretch compared with no-stretch mice (p = 0.04; Fig. 3B, Table 1). This suggests that stretching may counteract CD8^+^ T cell impairment and allow adaptive cytotoxic immune responses against the tumor to take place. We further tested this hypothesis by measuring intracellular cytokines produced by CD8^+^ T cells isolated from the tumor-draining (axillary) lymph node and stimulated with PMA/ionomicin *in vitro*. We hypothesized that T-cells from stretched mice would have a stronger effector function, manifested as an increase in inflammatory cytokines within CD8^+^ T cells in response to stimulation. Although there were no significant differences in TNF-α^+^ and INF-γ^+^ lymphocytes between stretch and no stretch, IL-2^+^CD8^+^ lymphocytes were significantly more abundant in the stretch group (p = 0.04; Fig. 3C, Table 1), which supports an effect of stretching on T-cell activation.

**Figure 4:**
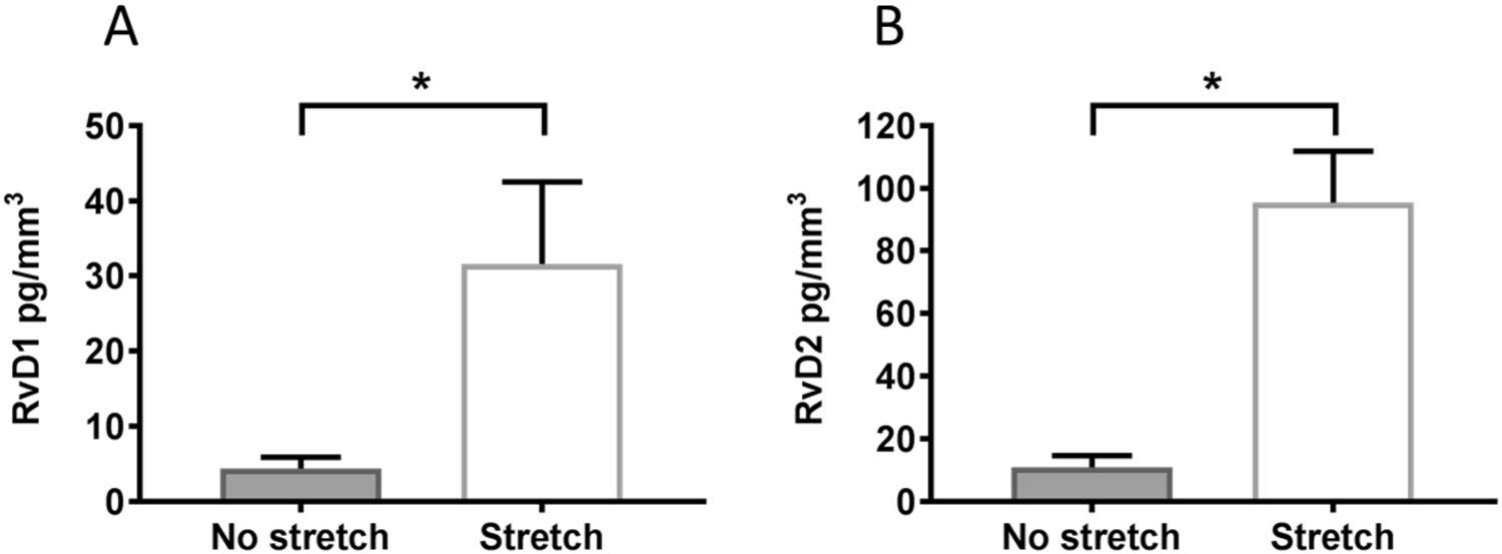
Stretching increases pro-resolving mediators in p53/PTEN−/− mouse breast cancer model: (**A,B**) SPMs measured within tumors by ELISA. A: Resolvin-D1. B: Resolvin-D2 (Median ± SE, N = 10/group) *p < 0.05.

Although inflammation is an important part of cytotoxic immunity, it can also be detrimental as chronic uncontrolled inflammation in the tumor microenvironment can promote tumor growth^28^. For this reason, we investigated the effect of stretching on tumor levels of lipid-derived Specialized Pro-Resolution Mediators (SPMs) RvD1 and RvD2 which promote the natural resolution of inflammation^29^, and were recently shown to suppress tumor growth in murine models^30^. Furthermore, our previous studies showed that stretching increased the production of RvD1 in the carrageenan inflammation model^23^. We therefore measured levels of SPMs within tumors and found that levels of RvD1 and RvD2 were both significantly greater in stretch vs. no-stretch mice (p = 0.04, Fig. 4). Together, our results suggest that pro-resolution mechanisms may act in concert with increased cytotoxic immunity to reduce the growth of tumors in response to stretching.

## Discussion

To our knowledge, this is the first report of reduced cancer growth in response to stretching. In addition, our data suggest that stretching may impact two potentially related mechanisms relevant to cancer: immune cell exhaustion and inflammation resolution. Inflammation is a double edge sword in cancer: although it is an essential component of all immune responses, it needs to be limited both in location and duration. Moreover, it is becoming clear that mechanisms to dampen inflammation are built-in to the inflammatory response itself. INF-γ exemplifies this duality since, in addition to being a classic inflammatory cytokine, it has recently been shown to contribute to the pro-resolving action of RvE1^31^. Sulciner *et al*. recently reported that the injection of SPMs (RvD1, RvD2 and RvE2) suppressed the growth of tumors by stimulating the clearing of tumor apoptotic debris^30^. It is therefore an interesting possibility that stretching could play a role in keeping inflammation under control while not suppressing the natural evolution of the cytotoxic immune response against the cancer cells, and even “rescuing” the cytotoxic function of exhausted T-cells. To our knowledge there has been no previous research linking inflammation resolution to T-cell recovery from immune exhaustion. Our results therefore support a potentially important link between these two areas of inquiry.

A limitation of our study is that, due to the small size of tumors, we were not able to measure intracellular cytokine production in the tumors themselves. However, we did examine CD8+ T-cells from the tumor-draining axillary lymph node, where we found an increase in intracellular IL-2 production, one of the most important cytokines involved in cytotoxic T-cell activation and clonal proliferation. Another limitation of the study is that we did not explore NK phenotype or function, which is also a key innate defense mechanism against tumors. Further studies in larger tumors or a different tumor model will be needed to examine these questions.

One key consideration is that, like exercise, our active stretching model is a complex intervention. In addition to direct stretching of tissue surrounding the tumor and draining lymph node, our intervention includes other components such as muscle activity and stress which are not present in the control group. While our method of stretching does not fully suspend the animal, since its front feet are allowed to grip a bar of the cage, some stress inevitably results from being restrained by the tail, and muscle activity is required to maintain the stretched posture; in the no stretch group, we did not hold the mice by the tail, as they have a tendency to pull away which can cause inadvertent stretching of tissues. To address these multiple factors in our stretching model, we previously compared active stretching, passive stretching under anesthesia and anesthesia alone without stretching in a carrageenan inflammation model. Both active and passive stretching had similar effects compared with anesthesia alone, and there was no significant difference between the effects of active and passive stretching^23^. This result demonstrated a mechanical effect of stretching that was not due to stress or muscle activity (since these were identical in the two anesthetized groups). This was further supported by an experiment showing the local release of RvD1 within connective tissue in response to stretching *ex vivo*^23^, which is independent of blood and lymphatic flow, stress and muscle activity. In the current study, passive stretching was not feasible in our tumor models, since it would have involved daily anesthesia for one month. We therefore cannot rule out that mild stress may have contribute to the beneficial effect of stretching in the cancer model, similar to the beneficial effect of acute stress during exercise^9^, perhaps in conjunction with tissue stretching.

Whether or not stress is involved, the possibility that stretching is having a local, mechanically-driven effect on the tumor stroma and immune environment is of interest given the current growing field of cancer mechanobiology^32,33^. Importantly, however, it will be critical to test whether the mechanical action of stretching tissues could also increase the likelihood of metastatic seeding. Mechanical compression of tumors during massage has been linked to the presence of labeled epithelial cells in sentinel axillary nodes in patients with breast cancer who underwent breast massage prior to the procedure^34^. Because stretching tissues in one dimension likely results in some amount of perpendicular compression, this question is relevant to our model and will need to be examined. Another important consideration is that, in this study, stretching was performed for 10 minutes once a day at ~25% strain, and we do not know whether a lower “dose” of stretching could be equally effective. Determining the minimum effective amplitude, duration and frequency of stretching in animals will be important before translational studies in humans can be undertaken. Meanwhile, it is interesting to note that the simultaneous extension of fore and hind-limbs in our stretching method is a core component of yoga poses that are commonly used in integrative oncology programs for cancer patients and are well tolerated^18^. These yoga poses therefore could be a good starting point for developing a stretching protocol to be tested in humans, once preclinical dosing and safety studies have been completed.

In conclusion, our results demonstrate a 52% reduction of mammary tumor growth over one month in mice undergoing stretching for 10 minutes once a day without any other form of therapy. The potential clinical significance of our results lies in the possibility of developing a method of gentle stretching that could be well tolerated and testable in humans for primary or secondary cancer prevention, or in conjunction with cancer treatment. Although the mechanisms underlying the beneficial effects of stretching in our mouse breast cancer model remain to be elucidated, our results point to a possible link between inflammation resolution and immune exhaustion mechanisms that could be important in basic cancer biology.

## Methods

Protocols were approved by the Brigham and Women’s Hospital (BWH) IACUC committee (#04995) and all methods and protocols were performed in accordance with BWH institutional guidelines. Materials, data and associated protocols will be made available without undue qualifications in material transfer agreements.

### Tumor cell preparation and injection

For the p53PTEN−/− breast cancer model, six-week old female FVB mice (Jackson Laboratory, Bar Harbor, ME) (N = 66) underwent bilateral tumor cell injection. Primary mouse mammary tumor cells were derived from tumors generated from *K14-Cre::Pten*^*f/f*^*;Trp53*^*f/f*^ by crossing *K14-Cre::Pten*^*f/f*^ ^35^ with *Trp53*^*f/f*^. Mice were of pure FVB background, thus allowing transplantation into syngeneic commercial FVB mice. Single tumor cell suspensions were prepared in 40% matrigel in DMEM. 100,000 tumor cells in 100 µl were injected into the 3^rd^ mammary fat pad bilaterally.

### Stretching

Before injection, mice were randomized to stretch or no-stretch for 10 minutes once/day, for four weeks. Mice were stretched as previously described^23^ by lifting them by the base of the tail until reaching ~45° angle to horizontal (Fig. 1). With minimal habituating, mice could hold this position comfortably for 10 minutes without struggling or vocalizing. Mice randomized to no stretch were taken out of their cages and placed on a table, but not restrained or lifted, for the same amount of time as the stretch group.

### Tumor volume measurement

The main outcome was tumor volume measured immediately after euthanasia at the end of week 4. In a subset of mice (N = 46), tumor volume was also measured under anesthesia at weeks 2 and 3. All volume measurements were performed *in situ* using calipers by a blinded investigator. Tumor weight was measured on excised tumors *post mortem*. No mice were euthanized before 4 weeks (i.e. no tumor became ulcerated, and no animal developed evidence of pain or distress). Individual tumor volume was calculated as π/6 × L × W^2^ ^36^. Maximum tumor volume was 1.2 cm^3^. Total tumor burden was calculated as the sum of tumor volumes on right and left sides.

### Carrageenan inflammation

C57 B6 male mice between 8–9 weeks’ old were used for this particular set of experiments. The lumbar subcutaneous space, 0.5 cm lateral of the spine at the L3 level, was injected with 50 µl of 2% carrageenan (Sigma), diluted in sterile PBS. Animals were randomly assigned to stretch or no stretch and treated twice a day for 3–5 days. Mice were euthanized and lesions were harvested, minced in PBS using a scalpel and cells were isolated and analyzed for measurement of cytokine production by flow cytometry.

### Flow cytometry

Tumors or lymph nodes were harvested and minced in 5% FBS-DMEM, using a syringe plunger. Isolated cells were counted using an automated cell counter TC20 (Biorad, CA). For surface receptors, cells at 1 × 10^6^/ml were stained with a mix of mouse monoclonal antibodies: CD3-FITC, TCR-FITC, anti-PD-1-PE, anti-CD8-PE-Cy7, anti-CD4-PE-Cy7, anti-CD45-APC-Cy7 (Biolegend, San Diego CA). Stained cells were examined using a FACSCanto II Flow Cytometer (BD Biosciences, San Jose, CA) with FlowJo single cell analysis software. For measurement of cytokine production, lymph node cells were incubated in 96-well plates, at 37 °C with a leukocyte activation cocktail consisting of BD GolgiPlug™ polyclonal cell activation mixture containing phorbol ester, 50 ng/ml PMA (Phorbol 12-Myristate 13-Acetate), 1 μg/ml ionomycin and 1 μg/ml protein transport inhibitor BD GolgiPlug™ (Brefeldin A), for 5 h. PMA and ionomycin were used in this experiment to boost cytokine production in functionally exhausted T-cells^37^. After incubation, cells were washed and prepared for surface and intracellular staining. Analysis of cytokine expression was performed on TCR+ CD8+ cells. Cytokine production was evaluated through intracellular staining by using the Citofix/Cytoperm Plus kit (Pharmingen) according to the manufacturer’s instructions. Surface-stained, fixed and permeabilized cells were incubated with the antibody of interest for cytokine detection: IL-2, IFN-γ and TNF-α. For the carrageenan experiment intracellular INF-γ was measured on CD4+ T cells.

### Soluble cytokine and Resolvin measurements

For secreted cytokines, supernatants from centrifugation of minced tumors were analyzed by a bead-based multiplex assay LEGENDPlex (BioLegend) for TH1/TH2 cytokine panel, according to manufacturer instructions using a FACSCanto II Flow Cytometer (BD Biosciences, San Jose, CA). Samples were analyzed using BioLegend’s LEGENDplex TM Data Analysis Software. Tissue levels of RvD1 and RVD2 were measured using an ELISA kit following the manufacturer’s instructions (Cayman Chemical, Ann Arbor, Michigan).

### Gene expression analysis

Gene expression analyses were performed as previously described^38^. In brief, RNA was isolated from bulk tumor fragments, and sequenced on an Ion Torrent Proton platform (Thermo Fisher) using a mouse-specific Ion AmpliSeq Custom Panel designed to target 3,827 cancer-related genes. Counts were generated using Torrent Suite and AmpliSeqRNA analysis plugin (Thermo Fisher). For GSEA, we used the MSigDB Hallmarks v6.1 dataset with weighted enrichment statistic and signal-to-noise metric for ranking genes.

### Statistical methods

Outcome measures were compared between stretch and no-stretch groups using two-sample t-tests for normally distributed measures and Exact Wilcoxon Rank Sum tests for those with skewed distributions or outliers present. Welch’s t-tests were used when group variances were unequal. Mixed model repeated measures analysis (SAS, PROC MIXED) was used to evaluate group differences in tumor growth. Significance was determined based on α = 0.05. All flow cytometry results represent the sum of right and left sides. Outcomes adjusted for individual tumor volume (cytokines, SPMs) are reported as the average of both sides.
