# Author Correction: Stretching Reduces Tumor Growth in a Mouse Breast Cancer Model

**DOI:** 10.1038/s41598-018-35364-w

**Published:** 2018-11-16

**Authors:** L. Berrueta, J. Bergholz, D. Munoz, I. Muskaj, G. J. Badger, A. Shukla, H. J. Kim, J. J. Zhao, H. M. Langevin

**Affiliations:** 1 Osher Center for Integrative Medicine, Division of Preventive Medicine, Brigham and Women’s Hospital, Harvard Medical School, Boston, USA; 2 Dana Farber Cancer Institute, Institute, Department of Cancer Biology, Boston, USA; 3 Harvard Medical School Department of Biological Chemistry and Molecular Pharmacology, Boston, USA; 4 University of Vermont Department of Medical Biostatistics, Burlington, USA; 5 University of Vermont Cancer Center, Burlington, USA

Correction to: *Scientific Reports* 10.1038/s41598-018-26198-7, published online 18 May 2018

The Acknowledgements section in this Article is incomplete.

“We thank Sara Olenich, Kayley Abell-Hart and Thanh Von for technical assistance. This study was funded by the Osher Foundation, and in part by NCCIH RO1 AT009267.”

should read:

“We thank Sara Olenich, Kayley Abell-Hart and Thanh Von for technical assistance. This study was supported by the Osher Foundation (H.M.L.), NCCIH RO1 AT009267 (H.M.L.), Susan G. Komen Foundation PDF16376814 (J.B.), National Institutes of Health (NIH) R35 CA210057 (J.J.Z.) and Breast Cancer Research Foundation (J.J.Z.).”

